# Variability of serum CYFRA 21 − 1 and its susceptibility to clinical characteristics in individuals without cancer: a 4-year retrospective analysis

**DOI:** 10.1186/s12890-023-02650-x

**Published:** 2023-09-13

**Authors:** Asami Minamibata, Yoshihito Kono, Taichiro Arimoto, Yoshinori Marunaka, Koichi Takayama

**Affiliations:** 1 Medical Research Institute, Kyoto Industrial Health Association, 67 Kita-Tsuboicho Nishinokyo Nakagyo-ku, Kyoto, 604-8472 Japan; 2 Department of Pulmonary Medicine, Graduate School of Medical Science, Kyoto Prefectural University of Medicine, Kyoto, Japan

**Keywords:** CYFRA 21 − 1, Keratin, Age, Smoking, Screening, Lung cancer

## Abstract

**Background:**

CYFRA 21 − 1 is a useful marker for diagnosing and monitoring lung cancer. However, its stability remains unclear. Moreover, while its applicability to screening is now being investigated, CYFRA 21 − 1 levels in individuals without cancer, who are targets for cancer screening, have not yet been the focus of research. Therefore, the present study investigated variability in and the factors increasing serum CYFRA 21 − 1 levels.

**Methods:**

This retrospective study recruited 951 individuals undergoing annual medical examinations for six years. We used data obtained in the first four years. Variability in serum CYFRA 21 − 1 levels over a period of four years were investigated. CYFRA 21 − 1 was categorized as normal (≤ 3.5 ng/ml) or elevated (> 3.5 ng/ml). The rate of an elevated level in one visit and the transition from an elevated to normal level between visits were visualized. A multiple logistic regression model was used to study the relationships between the frequency of elevated CYFRA 21 − 1 levels and clinical characteristics, such as age, sex, body mass index, weight changes, and the smoking status.

**Results:**

Approximately 5% of subjects had elevated CYFRA 21 − 1 levels once in five tests over four years, while 15% had elevated CYFRA 21 − 1 levels once or more. Among subjects with elevated CYFRA 21 − 1 levels in one blood test, between 63 and 72% had normal levels in the next test. The median CYFRA 21 − 1 level in subjects with elevations in one blood test significantly decreased in the next test at all four time points. The frequency of elevated CYFRA 21 − 1 levels was associated with an older age [odds ratio (OR) = 6.99, 95% confidence interval (CI) = 3.01–16.2], current heavy smoking (OR = 3.46, 95% CI = 1.52–7.9), and weight loss (OR = 1.86, 95% CI = 1.07–3.24).

**Conclusions:**

Variability in and the factors increasing serum CYFRA 21 − 1 levels beyond the cut-off value need to be considered when interpretating CYFRA 21 − 1 test results. The future application of CYFRA 21 − 1 to lung cancer screening may require more than a single measurement.

## Introduction

Lung cancer is the leading cause of cancer death worldwide (18.0% of all cancer deaths) and caused an estimated 1.8 million deaths in 2020. The five-year survival rate of patients diagnosed with lung cancer between 2010 and 2014 was between 10 and 20% in most countries [1]. Although lung cancer generally has a poor prognosis, that of early-stage lung cancer is better and responds well to treatment. Two lung cancer screening trials, the National Lung Screening Trial [2] and the Nederlands-Leuvens Longkanker Screenings Onderzoek trial [3], showed that the screening of high-risk individuals with low-dose computed tomography (LDCT) may reduce lung cancer mortality, and this was attributed to detection in the early stages of disease. Therefore, the US Preventive Service Task Force recommends adults aged 50–80 years with a 20 pack-year smoking history who currently smoke or have quit within the past 15 years to undergo screening for lung cancer with LDCT [4]. The use of a single biomarker is not currently recommended for lung cancer screening. The combination of biomarkers, including CYFRA 21 − 1, has potential as a modality that will facilitate lung cancer screening [5, 6]. CYFRA 21 − 1, a fragment of cytokeratin 19, was identified as a sensitive marker for lung cancer in 1993 [7, 8] and is commonly used in the management of lung cancer. An elevated CYFRA 21 − 1 level has been proposed as a negative prognostic indicator in non-small cell lung cancer [9–11]. While many studies have investigated its clinical utility, the stability of CYFRA 21 − 1 levels remains unclear. Moreover, while the usefulness of combining CYFRA 21 − 1 with other biomarkers in cancer screening has been investigated, CYFRA 21 − 1 levels in individuals without cancer, who are targets for cancer screening, have not yet been the focus of research. Therefore, we used data collected on subjects without cancer to investigate variability in serum CYFRA 21 − 1 levels over a period of four years. We also examined the relationships between subject characteristics and the frequency of elevated CYFRA 21 − 1 levels.

## Methods

### Participants and study design

Data were extracted from subjects undergoing annual medical examinations at the Kyoto Industrial Health Association between April 2015 and March 2022. These data included a review of self-reported medical histories and the smoking status as well as the results of a physical examination, blood and urine tests, electrocardiogram, chest X-ray, abdominal ultrasound, upper gastrointestinal series or endoscopy, and an immunochemical fecal occult blood 2-day test. Tumor marker levels, including CYFRA 21 − 1 and carcinoembryonic antigen (CEA), were measured when requested. Every year, more than 20,000 individuals undergo a medical examination at the Kyoto Industrial Health Association. We extracted 1,059 individuals who underwent seven medical examinations, which included tumor marker measurements, for six consecutive years. Eighty-six individuals diagnosed with cancer by the most recent medical examination and twenty-two individuals with chest X-ray abnormalities in the most recent medical examination were excluded. We ultimately extracted 951 individuals and regarded data obtained in the first four out of six years as data for healthy individuals.

We used data obtained five times from each subject over a period of four years. Variability in serum CYFRA 21 − 1 levels over a period of four years was investigated and compared with that in CEA. CYFRA 21 − 1 and CEA levels were categorized as normal (≤ 3.5 ng/ml for CYFRA 21 − 1, ≤ 5.0 ng/ml for CEA) or elevated (> 3.5 ng/ml for CYFRA 21 − 1, > 5.0 ng/ml for CEA). The rate of an elevated level in one visit and the transition from an elevated to normal level between visits were visualized. We classified subjects into a frequently elevated or almost normal (once or no elevation) group according to whether subjects had elevated CYFRA21-1 levels twice or more over four years. We also examined the relationships between subject characteristics (including age, sex, body mass index (BMI), weight changes in four years, and the smoking status) and the frequency of elevated CYFRA 21 − 1 levels.

### Tumor marker measurements

Blood samples were obtained by peripheral venipuncture after fasting for more than 5 h. Serum samples were analyzed on a commonly available electrochemiluminescence immunoassay analyzer (Cobas e801; Roche Diagnostics) for CYFRA 21 − 1 and a chemiluminescence immunoassay analyzer (ADVIA Centaur XP; Siemens Diagnostics) for CEA. Cut-off values were set at 3.5 ng/ml for CYFRA 21 − 1 and 5.0 ng/ml for CEA according to the corresponding manufacturers’ suggestions and are clinically used in Japan.

### Statistical analysis

All data were expressed as numbers (percentages) for categorial variables or as medians and interquartile ranges (IQR). Wilcoxon’s signed-rank test was used to examine median changes in marker levels between visits. Differences in the characteristics of the two groups were tabulated using the chi-squared test for categorical variables and the Mann-Whitney U-test for continuous variables. The response variable was the presence or absence of a CYFRA 21 − 1 level > 3.5 ng/ml twice or more, and a multiple logistic regression model was used to identify adjusted relationships between explanatory variables and the response variable. Explanatory variables included age, sex, BMI, weight changes, and the smoking status. Odds ratios (OR) with 95% confidence intervals (CI) were used as a measure of the relationships between explanatory variables and the response variable. These statistical analyses were conducted with EZR software version 1.55 (Saitama Medical Center, Jichi Medical University, Saitama, Japan) [12]. The longitudinal reliability of CYFRA 21 − 1 and CEA was evaluated by two-way consistency average measures intraclass correlation coefficient (ICC) using R version 4.1.3. ICC values were calculated using log-transformed data. Based on the 95% CI of the ICC estimate, values less than 0.5, between 0.5 and 0.75, between 0.75 and 0.9, and greater than 0.9 were interpreted as poor, moderate, good, and excellent reliability, respectively [13]. *P*-values < 0.05 were considered to be significant.

## Results

**Fig. 1:**
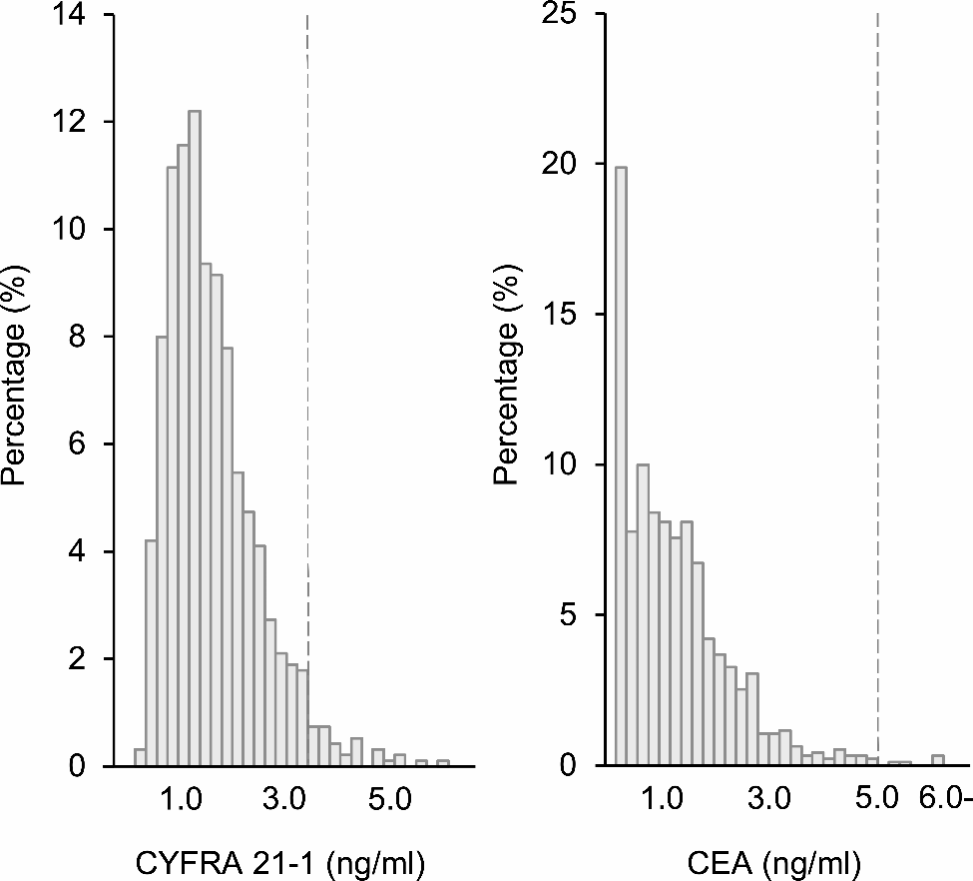
Distributions of CYFRA 21 − 1 and CEA levels in 2015

**Fig. 2:**
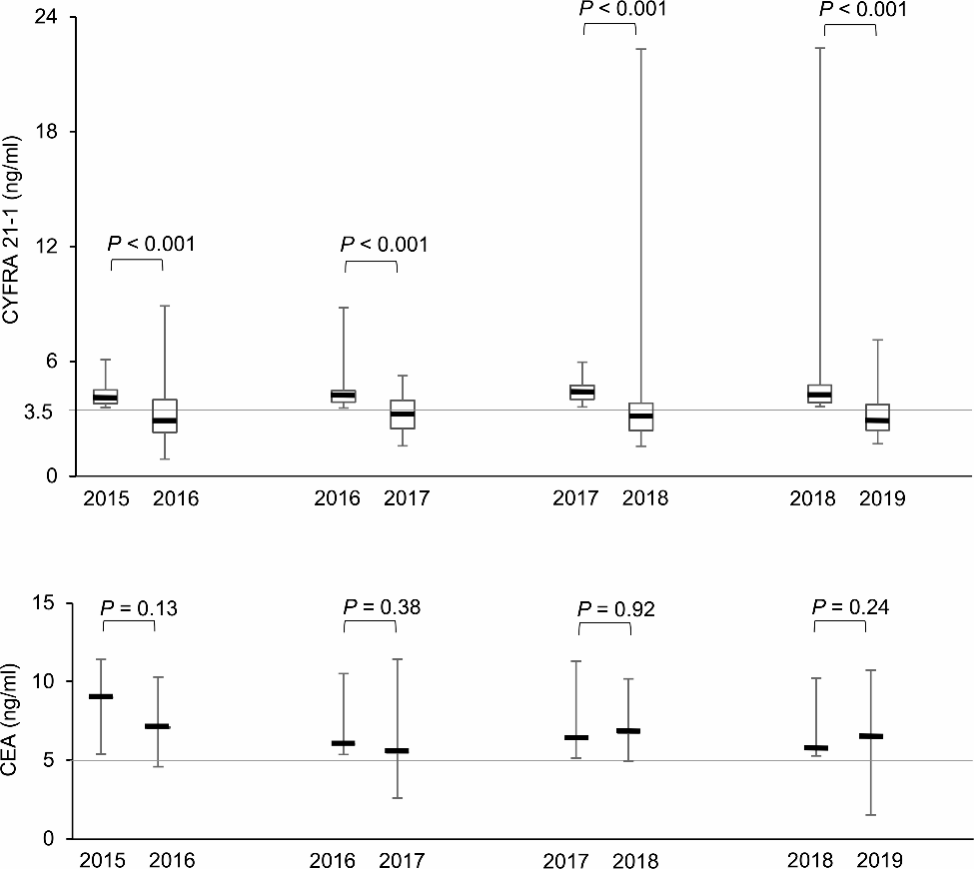
The transition of elevated CYFRA 21 − 1 and CEA levels between tests

A total of 951 subjects (737 males and 214 females) aged 29–80 years were included in the present study. The distributions of CYFRA 21 − 1 and CEA levels in 2015 are shown in Fig. 1. Median [IQR] CYFRA 21 − 1 and CEA levels were 1.6 [1.2–2.2] and 1.2 [0.6–1.8] ng/ml, respectively. Among 951 subjects, the number of subjects with CYFRA 21 − 1 levels > 3.5 ng/ml and CEA levels > 5.0 ng/ml in 2015 were 33 (3.5%) and 5 (0.5%), respectively (Table I). Based on the 95% CI of the ICC estimate, the reliabilities of CYFRA 21 − 1 and CEA were good (ICC = 0.87, 95% CI = 0.85–0.88) and excellent (ICC = 0.98, 95% CI = 0.97–0.98), respectively. We investigated variability in tumor markers by calculating the frequency of elevated tumor marker levels being maintained in the next test. The transition of elevated CYFRA 21 − 1 and CEA levels is shown in Fig. 2. The percentages of subjects with elevated tumor marker levels that were maintained in the next test were 36, 37, 37, and 28% for CYFRA 21 − 1 and 60, 75, 83, and 71% for CEA over four years (Table II). Therefore, more than 60% of subjects with elevated CYFRA 21 − 1 levels in one blood test had normal levels in the next test. The median CYFRA 21 − 1 level in subjects with elevated levels in one blood test significantly decreased in the next test at all four time points. No significant differences were observed in CEA levels.

**Table I:** Marker levels and number of subjects with CYFRA 21 − 1 levels > 3.5 ng/ml and CEA levels > 5.0 ng/ml

		CYFRA 21 − 1	CEA
Median [IQR] (ng/ml)			
	In 2015	1.6 [1.2, 2.2]	1.2 [0.6, 1.8]
	In 2016	1.7 [1.3, 2.3]	1.1 [0.5, 1.7]
	In 2017	1.8 [1.3, 2.4]	0.9 [0.4, 1.6]
	In 2018	1.7 [1.3, 2.4]	1.1 [0.5, 1.7]
	In 2019	1.8 [1.3, 2.4]	1.0 [0.4, 1.7]
Number (%) that exceeded the cut-off value			
	In 2015	33 (3.5%)	5 (0.5%)
	In 2016	43 (4.5%)	8 (0.8%)
	In 2017	52 (5.5%)	6 (0.6%)
	In 2018	54 (5.7%)	7 (0.7%)
	In 2019	53 (5.6%)	5 (0.5%)

**Table II:** The transition of marker levels and percentage remaining above cut-off levels in the next test in subjects with elevated marker levels in one test

	2015	2016	2017	2018
CYFEA 21 − 1				
Median [IQR] (ng/ml)				
At the baseline	4.0 [3.8, 4.5]	4.2 [3.9, 4.5]	4.4 [4.0, 4.7]	4.2 [3.8, 4.8]
In the next test	3.0 [2.3, 4.0]	3.3 [2.5, 4.0]	3.1 [2.4, 3.8]	2.9 [2.4, 3.8]
Number (%) (> 3.5 ng/ml)				
At the baseline	33	43	52	54
In the next test	12 (36%)	16 (37%)	19 (37%)	15 (28%)
CEA				
Median [IQR] (ng/ml)				
At the baseline	9.0 [5.5, 9.3]	6.0 [5.6, 7.8]	6.4 [5.4, 7.7]	5.7 [5.5, 8.1]
In the next test	7.1 [4.7, 10]	5.5 [4.9, 7.2]	6.9 [5.7, 8.2]	6.4 [4.7, 7.5]
Number (%) (> 5.0 ng/ml)				
At the baseline	5	8	6	7
In the next test	3 (60%)	6 (75%)	5 (83%)	5 (71%)

**Fig. 3:**
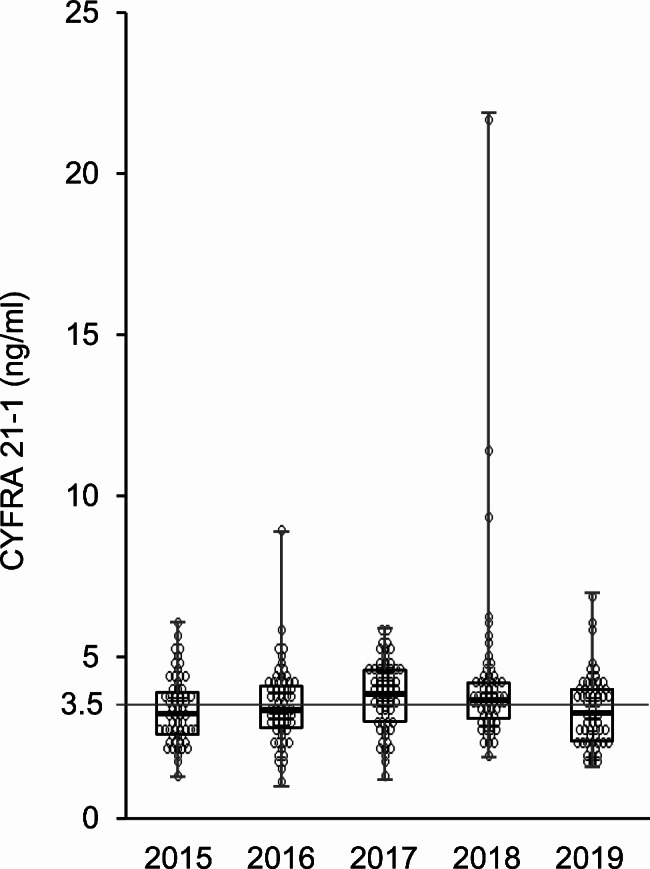
CYFRA 21 − 1 levels at each time point and their transition over four years in the group with elevated CYFRA 21 − 1 levels twice or more

**Fig. 4:**
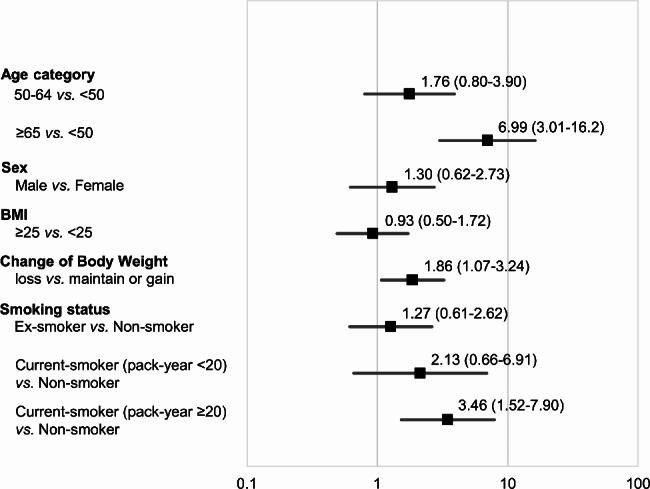
Results of a multivariable logistic analysis of factors associated with the frequency of elevated CYFRA 21 − 1 levels (> 3.5 ng/ml, twice or more)

Subject characteristics at the last test are shown in Table III. A total of 144 subjects (15.1%) had elevated CYFRA 21 − 1 levels in one or more of the five tests. In contrast, the rate of subjects with elevated CEA levels in one or more of the five tests was only 1.2%. To investigate the clinical characteristics of subjects with frequently elevated CYFRA 21 − 1 levels, subjects were categorized into two groups based on whether they had elevated levels twice or more in five tests. Regarding the group of subjects with elevated levels twice or more, CYFRA 21 − 1 levels at each time point and their transition over four years are shown in Fig. 3. The distribution of CYFRA 21 − 1 levels was close to the cut-off value (3.5 ng/ml). Subjects with elevated CYFRA21-1 levels twice or more were more likely to be older, current heavy smokers (20 pack-year or more), and have lost weight. The results of the multivariable logistic analysis of factors associated with the frequency of elevated CYFRA 21 − 1 levels (> 3.5 ng/ml, twice or more) are shown in Fig. 4. The frequency of elevated CYFRA 21 − 1 levels was associated with an older age (OR = 6.99, 95% CI = 3.01–16.2 for ≥ 65 years vs. <50 years), current heavy smoking (OR = 3.46, 95% CI = 1.52–7.9 for current smokers with a 20 pack-year or more smoking history vs. non-smokers), and weight loss in four years (OR = 1.86, 95% CI = 1.07–3.24 for weight loss vs. weight maintenance or gain).

**Table III:** Characteristics of subjects in the final test

		Total	Not frequently elevated	Frequently elevated	*p*-value
Number of subjects		951	892	59	
Sex (male, %)		77.5	77.5	78.0	1.000
Age at the baseline					
Median [IQR] (years)		54 [47, 61]	54 [47, 60]	61 [55, 67]	< 0.001
Categories (%)	< 50	31.4	32.5	15.2	< 0.001
	50–64	54.4	54.9	45.8	
	≥ 65	14.2	12.6	39.0	
BMI at the baseline					
Median [IQR]		23.1 [21.3, 25.2]	23.2 [21.4, 25.2]	22.0 [20.8, 25.1]	0.111
Categories (%)	< 25	73.2	73.2	72.9	1.000
	≥ 25	26.8	26.8	27.1	
Weight change in the follow-up period					
	Maintenance or gain	58.1	59.2	42.4	0.016
	Loss	41.9	40.8	57.6	
Smoking status at the baseline					
Categories (%)	Non-smoker	39.6	40.2	30.5	0.057
	Ex-smoker	40.7	40.8	39.0	
	Current (pack-year < 20)	7.1	7.1	6.8	
	Current (pack-year ≥ 20)	12.6	11.9	23.7	
Number of times (CYFRA 21 − 1 > 3.5 ng/ml)					
the rate of numbers (%)	0	84.9	90.5	0	< 0.001
	1	8.9	9.5	0	
	2	3.7	0	59.3	
	3	1.9	0	30.5	
	4	0.4	0	6.8	
	5	0.2	0	3.4	
Number of times (CEA > 5.0 ng/ml)					
the rate of numbers (%)	0	98.8	98.9	96.6	0.198
	1	0.6	0.6	1.7	
	2	0.1	0.1	0	
	3	0	0	0	
	4	0.2	0.2	0	
	5	0.3	0.2	1.7	

## Discussion

The present study examined variability in CYFRA 21 − 1 levels beyond the cut-off value and the factors increasing CYFRA 21 − 1 levels using data obtained from medical examinations over four years. Approximately 5% of subjects without cancer had elevated CYFRA 21 − 1 levels once in five tests over four years, while 15% had elevated CYFRA 21 − 1 levels once or more, which was > 10-fold higher than the rate for CEA (1.2%). The distribution of CYFRA 21 − 1 levels was closer to the cut-off level than that of CEA levels. Additionally, more than 60% of subjects with elevated CYFRA 21 − 1 levels in one test had a normal level in the next test. An older age, current heavy smoking, and weight loss were associated with the frequency of elevated CYFRA 21 − 1 levels (twice or more in five tests). To the best of our knowledge, this is the first study to examine CYFRA 21 − 1 levels in subjects without cancer over a period of four years. The results obtained provide novel information for use in analyses of the findings obtained in a physical examination of healthy individuals.

CYFRA 21 − 1 has been used regardless of sex, the smoking status, age, and other clinical characteristics. However, previous studies reported a relationship between CYFRA 21 − 1 levels and the smoking status [14, 15] or an older age [16]. In our previous study, an older age, current smoking, and low BMI were associated with high CYFRA 21 − 1 levels [17]. Previous studies only measured CYFRA 21 − 1 levels once. In contrast, the present study investigated the relationships between the frequency of elevated CYFRA 21 − 1 levels over a period of four years and clinical characteristics. An older age, current heavy smoking, and weight loss were associated with a higher frequency of elevated CYFRA 21 − 1 levels. CYFRA 21 − 1 is the soluble fragment of cytokeratin 19. Cytokeratin is widely referred to as keratin based on an internationally accepted consensus [18]; therefore, we use the term “keratin”. Keratins are major intermediate filament proteins that are selectively expressed in epithelial cells. They consist of type I (acidic intermediate filament proteins) and type II (basic intermediate filament proteins) encoded by 54 human keratin genes and are obligate non-covalent heteropolymers that include at least one type I and one type II keratin. A unique feature of keratins is that different keratin pairs are specifically expressed in epithelial cells. Many simple (single-layered) epithelia express variable levels of combinations of type II keratin 7 (K7) or K8 and type I K18, K19, and K20 [19]. K19 is found in the respiratory epithelium, gastrointestinal epithelia, ductal epithelia, and urothelium, and is occasionally present in the basal cells of non-keratinizing stratified squamous epithelia [20]. In cancer, K19 is expressed in most adenocarcinomas and squamous cell carcinomas of various organs, including the lung, stomach, colon, pancreas, ovary, and breast [20, 21]. Furthermore, the expression levels of intermediate filament proteins frequently increase with exposure to stress, such as shear stress, heat shock, toxins, aging, oxidative stress, or infection [22–24]. Similarly, K19 protein and messenger RNA levels were found to be significantly higher in middle-aged mice than in young mice after bone fracture and soft tissue trauma [25]. One of the major responses to stress is apoptosis, which leads to cell death by the caspase-dependent degradation of cellular proteins. During apoptosis, many intermediate filaments are cleaved by caspase. Apoptosis is one of the fundamental biological processes that are essential for manifold cellular functions in health and disease as autophagy and the unfolded protein response [26]. In lung cancer, CYFRA 21 − 1 was shown to be produced when K19 was cleaved by caspase 3 [27]. Additionally, recent studies on idiopathic pulmonary fibrosis (IPF) indicated the potential of CYFRA 21 − 1 as an important prognostic biomarker [28] and cleaved caspase-3 was associated with reduced lung function [26]. In IPF, the excessive apoptosis of alveolar epithelial cells and reduced sensitivity to apoptosis by fibroblasts and myofibroblasts were suggested to coexist and promote fibrosis [29], and senescence has also been shown to play a central role [30]. Senescence is one of the established causes of aging and aging-related disorders, and may be triggered by the multiple genetic changes induced by oxidative stress or damage to DNA or telomeres [31]. Aging lungs are more susceptible to injury than younger lungs, resulting in severe apoptosis [32]. Cigarette smoking, which is a major risk factor for IPF, similar to other lung diseases, is known to increase oxidative stress, which leads to senescence and the activation of pro-inflammatory pathways in the airway epithelium [33]. Collectively, these findings suggest that the expression level of K19 increases with exposure to stress, and CYFRA 21 − 1 is produced when K19 is cleaved during apoptosis. CYFRA 21 − 1 levels may increase even in individuals without cancer. Therefore, CYFRA 21 − 1 may be released during the apoptosis of epithelial cells in lungs exposed to stress, such as cigarette smoke and aging. In individuals exposed to stress, the extent of exposure and the degree of apoptosis varies with time. This variability may affect CYFRA 21 − 1 levels. The present study showed that the frequency of elevated CYFRA 21 − 1 levels was associated with weight loss. Muscle wasting may also simultaneously occur. K19 is expressed in muscle [19]. Aging is associated with progressive declines in muscle mass and quality, which is a condition called sarcopenia. Muscle apoptosis was previously shown to be associated severe sarcopenia, but not aging [34]. Muscle apoptosis may be responsible for the relationship between CYFRA 21 − 1 and weight loss. However, limited information is currently available on the relationship between CYFRA 21 − 1 and weight loss, and body weight is affected by many factors both consciously and unconsciously. Therefore, this relationship is subject to debate. Since many factors may also change CYFRA 21 − 1 levels, this issue warrants further study.

The present results showed that the distribution of CYFRA 21 − 1 levels was closer to the cut-off level than that of CEA levels. CYFRA 21 − 1 levels varied more beyond the cut-off level than CEA levels. CEA was distributed around a lower level. CEA is a cell surface glycoprotein that is anchored on the cellular membrane. Some membrane-bound CEA is cleaved from the cell membrane and secreted into blood. CEA mediates cell-cell adhesion and modulates cellular processes [35]. It is expressed by normal epithelial cells; however, CEA levels are markedly lower in serum than in cancer cells. High CEA levels have been reported in lung cancer and many other cancers as well as in benign lung diseases, such as chronic obstructive pulmonary disease and interstitial lung disease [36]. The mechanisms underlying elevated CEA levels in benign lung disease currently remain unclear. Only a few studies have examined the distributions of CEA and CYFRA 21 − 1 levels in healthy individuals. A previous study showed that median CEA levels were 0.60 ng/ml in healthy individuals vs. 1.50 ng/ml for benign lung diseases, while median CYFRA 21 − 1 levels were 1.19 ng/ml in healthy individuals vs. 1.17 ng/ml for benign lung diseases [37]. Another study reported CEA levels of 1.27 ng/ml in healthy individuals vs. 2.18 ng/ml for benign lung diseases and CYFRA 21 − 1 levels of 2.10 ng/ml in healthy individuals vs. 2.21 ng/ml for benign lung disease [38]. These studies included different types of lung diseases, subjects, and definitions of healthy individuals. Nevertheless, CEA levels in healthy individuals were distributed at a lower level, suggesting the poor specificity of CYFRA 21 − 1. Prior to the future application of CYFRA 21 − 1 to cancer screening, cut-off values need to be set for different groups in consideration of factors, such as age.

There are several limitations that need to be addressed. There are potential issues regarding the validity and integrity of medical histories and the smoking status. We were unable to evaluate the accuracy of self-reports on smoking habits and medical histories. Furthermore, some subjects may have had an undiagnosed lung disease. Although we examined medical histories and chest X-ray two years after the data period examined, there may have been individuals with early-stage lung cancer. Nevertheless, our large sample size may have minimized these effects. Another limitation is that the detection systems used at different times may have affected reliability. Moreover, we analyzed data from a single center in Japan. Therefore, the results obtained may not be transferrable to other races. A multicenter analysis of a large sample size is needed to identify the factors affecting tumor markers.

In conclusion, an older age, current heavy smoking, and weight loss were associated with the frequency of elevated CYFRA 21 − 1 levels. CYFRA 21 − 1 levels highly varied beyond the cut-off level. An older age and heavy smoking are major risk factors for lung cancer. Regarding the future application of CYFRA 21 − 1 levels to screening for lung cancer, variability in and factors increasing CYFRA 21 − 1 levels need to be considered and a single measurement of CYFRA 21 − 1 may be insufficient.

## Data Availability

The datasets used and/or analyzed during the present study are available from the corresponding author upon reasonable request.

## List of abbreviations

BMI: Body mass index
CI: Confidence interval
CEA: Carcinoembryonic antigen
IPF: Idiopathic pulmonary fibrosis
ICC: Intraclass correlation coefficient
IQR: Interquartile ranges
K19: Keratin 19
LDCT: Low-dose computed tomography
OR: Odds ratio

## Declarations

Since this was a retrospective study, informed consent was waived by the local research Ethics Committee of Kyoto Industrial Health Association. Prior to the medical examination, subjects were given the option to opt out of providing their data for medical research and were informed that they may withdraw at any time. Written information on the present study was provided on the website of the Kyoto Industrial Health Association.
